# Early Pregnancy Immune Signatures May Distinguish Aneuploid Miscarriage from Euploid Pregnancy Loss and Live Birth

**DOI:** 10.3390/ijms27062823

**Published:** 2026-03-20

**Authors:** Margarita Ruseva, Dimitar Parvanov, Rumiana Ganeva, Maria Handzhiyska, Jinahn Safir, Lachezar Jelezarsky, Stefka Nikolova, Dimitar Metodiev, Maria Pancheva, Maria Serafimova, Blaga Rukova, Rada Staneva, Georgi Stamenov, Savina Hadjidekova

**Affiliations:** 1 Research Department, Nadezhda Women’s Health Hospital, 1373 Sofia, Bulgaria; margarita.ruseva@gmail.com (M.R.); rum.ganeva@gmail.com (R.G.); mariavh@abv.bg (M.H.); jinahn.safir@gmail.com (J.S.); jelezarsky@gmail.com (L.J.); 2 Embryology Department, Nadezhda Women’s Health Hospital, 1373 Sofia, Bulgaria; stefka.v.nikolova@gmail.com; 3 Pathology Department, Nadezhda Women’s Health Hospital, 1373 Sofia, Bulgaria; dr.dmetodiev@yahoo.com; 4 Genetics Department, Nadezhda Women’s Health Hospital, 1373 Sofia, Bulgariasvhadjidekova@medfac.mu-sofia.bg (S.H.); 5 Department of Medical Genetics, Faculty of Medicine, Medical University–Sofia, 1431 Sofia, Bulgaria; blagarukova@yahoo.com (B.R.);; 6 Obstetrics & Gynecology Department, Nadezhda Women’s Health Hospital, 1373 Sofia, Bulgaria

**Keywords:** early pregnancy, peripheral blood immune cells, miscarriage, aneuploidy, T helper cells, basophils

## Abstract

Pregnancy loss affects ~15% of couples and often results from embryonic chromosomal abnormalities. Early peripheral biomarkers that signal abnormal development could improve counseling and clinical decision-making. Here, we analyzed early-pregnancy peripheral blood from patients who conceived via assisted reproduction without preimplantation aneuploidy testing. Samples were collected ≤12 weeks’ gestation for complete blood counts with differentials and multiparameter flow cytometry to quantify major lymphocyte subsets (total T, B, cytotoxic T cells, T helpers (Th), Th1, Th2, Th9, Th17, and regulatory T cells (Treg)). Participants were followed until pregnancy resolution (live birth, euploid or aneuploid miscarriage), and immune profiles were compared by outcome using the Kruskal–Wallis test. Exploratory discriminative analyses were performed with significantly different immune cell quantities. Basophils were highest in the aneuploid miscarriage group (n = 26), distinguishing them from both euploid miscarriage (n = 27) and live birth (n = 91). Th9 cells were lower in aneuploid miscarriages compared to euploid miscarriages. Th17 levels were higher in live births compared with both miscarriage groups. Additional aneuploidy-type-specific immune differences were observed. These alterations may reflect maternal immune recognition of a non-viable conceptus and localized immune activation at the fetal–maternal interface. If validated in larger cohorts, these early peripheral markers may help identify pregnancies at risk for miscarriage, particularly those involving chromosomal abnormalities.

## 1. Introduction

Pregnancy loss is one of the most frequent complications of human reproduction, affecting approximately 10–15% of clinically recognized pregnancies and up to 30% of biochemical pregnancies [1,2,3]. Beyond its physical and hormonal consequences, miscarriage has a profound emotional and psychological impact on affected couples, often associated with anxiety, depression, and post-traumatic stress [4]. The multifactorial etiology of pregnancy loss includes anatomical, endocrine, infectious, immune, and genetic causes [1,5]. Among these, chromosomal abnormalities in the embryo account for approximately 50–60% of spontaneous miscarriages during the first trimester [1,3,5,6].

Despite substantial progress in reproductive medicine and assisted reproductive technologies (ART), predicting which pregnancies are destined to fail remains challenging. Current early pregnancy monitoring primarily relies on clinical parameters, ultrasound measures and serum markers such as beta human chorionic gonadotropin (β-hCG) or progesterone, which offer predictive accuracy for embryonic viability in general but not chromosomal status [7,8]. Standard early prenatal screening for genetic anomalies includes a non-invasive prenatal test (NIPT), nuchal translucency measurement, and first-trimester serum markers (PAPP-A and β-hCG) [9]. These modalities have important limitations as they screen for only a small subset of common chromosomal aneuploidies and fail to detect most genetic disorders, including balanced chromosomal rearrangements, mosaicism, and many structural anomalies [10]. Their utility is further constrained by narrow gestational timing windows (usually 11–13 gestational weeks) and biological or technical factors that affect accuracy, e.g., NIPT relies on the proportion of fetal DNA in the maternal blood [11]. Importantly, all are screening rather than diagnostic tests; normal results do not exclude significant fetal abnormalities, and abnormal findings require confirmation with invasive diagnostic testing [12]. Identifying reliable early biomarkers that reflect abnormal embryonic development is crucial as it could improve clinical decision-making, allowing for timely intervention, personalized care, and more accurate counseling.

Extensive evidence suggests that maternal immune tolerance plays a pivotal role in the successful implantation and continuation of pregnancy [13]. Dysregulation in the balance between pro- and anti-inflammatory immune responses, or in the proportions of specific lymphocyte subsets, has been implicated in recurrent pregnancy loss and implantation failure [14]. For instance, altered ratios of T helper (Th1/Th2) and regulatory T cells (Treg), and deviations in natural killer or cytotoxic T cell activity, have been observed in pregnancy complications [13,15]. However, data focusing specifically on early immunological differences between pregnancies destined for chromosomally abnormal miscarriage, euploid miscarriage, and successful live birth remain limited.

The present study aimed to address this knowledge gap by characterizing the peripheral immune profiles of women during early pregnancy following ART conception. Specifically, we sought to compare quantitative and phenotypic differences in peripheral blood immune cell populations between women who subsequently experienced miscarriage due to chromosomal abnormalities, those with euploid miscarriage, and those who carried to term. Identifying distinct immune signatures associated with genetically abnormal gestations could provide novel insights into the maternal recognition of aneuploid embryos and lead to potential early biomarkers of non-viable pregnancies.

## 2. Results

### 2.1. Participant Characteristics

The current study was approved by the Nadezhda Women’s Health Hospital Ethics Committee (protocol code 7A/13012023). Female patients who conceived through assisted reproduction without preimplantation genetic testing were enrolled after signing informed patient consent. Among the enrolled cohort, 53 women experienced miscarriage, while 91 pregnancies resulted in live birth (Table 1). According to the inclusion criteria, all participants provided peripheral blood samples during the first trimester of pregnancy (≤12 weeks of gestation). As reflected in Table 1, the median sampling time was 6–7 weeks across groups, corresponding to the timing of the clinical pregnancy confirmation appointment. The majority of samples were obtained in this period, with 70% of participants providing blood samples before 8 weeks of gestation (Table S1). Importantly, gestational age at sampling did not differ significantly between the pregnancy outcome groups (Table 1).

In patients who experienced pregnancy loss, the miscarriages occurred between 7 and 14 weeks’ gestation. The majority of losses occurred during the early first trimester before the 9th gestational week. Pregnancies that resulted in live birth were delivered between 37 and 41 weeks of gestation (Table S1). Of the miscarriage cases, 26 (49%) were classified as aneuploid based on cytogenetic testing of products of conception. Among these, 14 cases revealed trisomies involving chromosomes 2, 7, 9, 10, 13, 15, 16, and 22. Four cases involved monosomies (chromosomes 21 and X), three showed high-grade mosaicism, two exhibited structural chromosomal rearrangements, and four demonstrated multiple aneuploidies within the same sample. The remaining 27 losses were classified as euploid. Miscarriages occurred between 7 and 14 weeks’ gestation. Patients who miscarried were slightly older than those with live births (39.4 ± 6.2 vs. 38.2 ± 6.9 years), although this difference was not statistically significant (*p* > 0.05). The age of participants across the entire cohort ranged from 23 to 47 years.

To provide a comprehensive overview of the study population, detailed individual-level information, including participant age, gestational age at sampling, timing of miscarriage or delivery, and pregnancy outcome, is provided in the Supplementary Materials (Table S1).

### 2.2. Peripheral Blood Cell Counts

**Figure 1:**
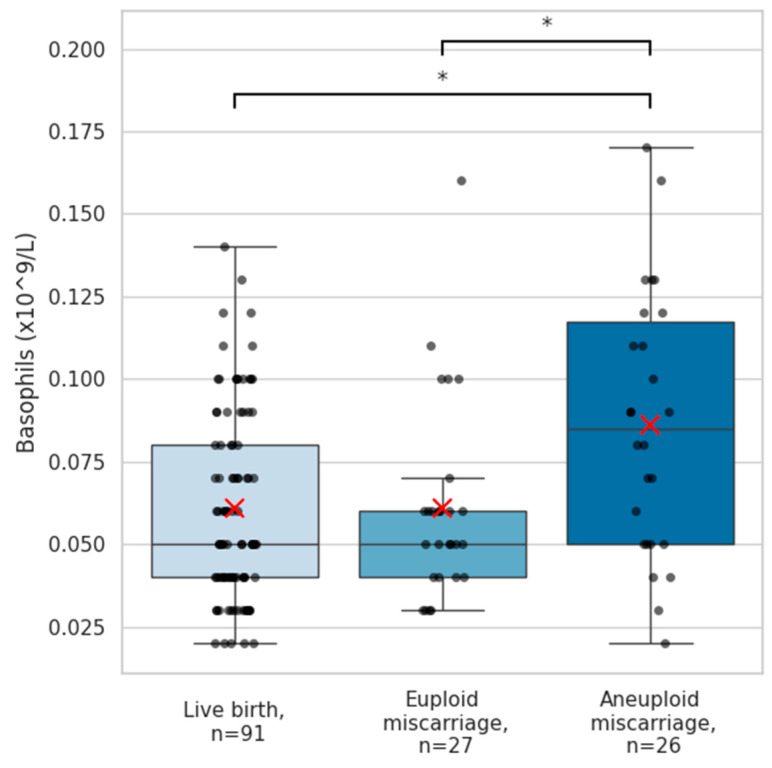
Basophil counts according to pregnancy outcome. Box-and-whisker plots depicting circulating basophil concentrations (×10^9^/L) in pregnancies resulting in live birth (n = 91), euploid miscarriage (n = 27), and aneuploid miscarriage (n = 26). Black dots represent individual samples; red “×” marks indicate group means. Group differences were assessed using an independent-samples Kruskal–Wallis test (overall *p* = 0.009). Post hoc pairwise comparisons with Bonferroni correction revealed significantly higher basophil levels in aneuploid miscarriages compared with live births (*p* = 0.018) and euploid miscarriages (*p* = 0.003). The asterisk “*” denotes significant difference between groups.

**Figure 2:**
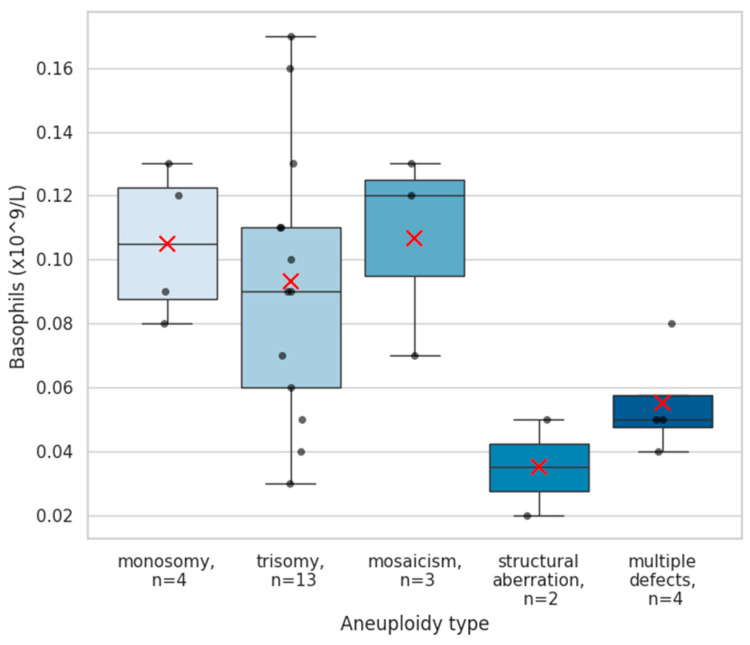
Basophil levels according to aneuploidy subtype. Boxplots depict circulating basophil concentrations (×10^9^/L) across aneuploid miscarriage subclasses, including monosomies (n = 4), trisomies (n = 13), mosaic aneuploidy (n = 3), structural chromosomal aberrations (n = 2), and cases with multiple aneuploid defects (n = 4). Individual patient values are shown as black dots, and red “×” marks denote group means. Among aneuploid pregnancies, the highest basophil counts were observed in trisomy cases, with similarly elevated levels in monosomic and mosaic losses, whereas samples with structural aberrations or multiple defects exhibited comparatively lower basophil concentrations.

A comprehensive complete blood count (CBC) analysis with differential was performed, including red blood cell count, total leukocyte counts and major leukocyte subpopulations (lymphocytes, neutrophils, eosinophils, and monocytes), as well as platelet counts. Across pregnancy outcome groups, no statistically significant differences were found in red blood cell, platelets or total leukocyte counts, nor in these major leukocyte subpopulations (all *p* > 0.05, Table A1). In contrast, basophil levels differed notably between pregnancy outcome groups. Basophils were significantly elevated among aneuploid miscarriage patients (median 0.09 [0.07] × 10^9^/L) compared with women with subsequent live births (0.05 [0.04] × 10^9^/L; *p* = 0.018) and those with euploid miscarriage (0.05 [0.02] × 10^9^/L; *p* = 0.003) (Figure 1). The basophil reference interval across all trimesters is 0.0–0.1 × 10^9^/L [16]. Basophil levels exceeded the upper limit of this range in 6 of 91 women (6.6%) with subsequent live births, in 2 of 27 patients (7.4%) who experienced a euploid miscarriage, and in 9 of 26 (34.6%) with an aneuploid miscarriage. When stratified by aneuploidy subtype, the highest basophil quantities were observed in cases with trisomies, followed by monosomies and mosaicism, which displayed similarly elevated distributions. In contrast, pregnancies affected by structural chromosomal aberrations or multiple concurrent defects showed comparatively lower basophil levels (Figure 2).

### 2.3. Flow Cytometry Immune Profiling

**Figure A1:**
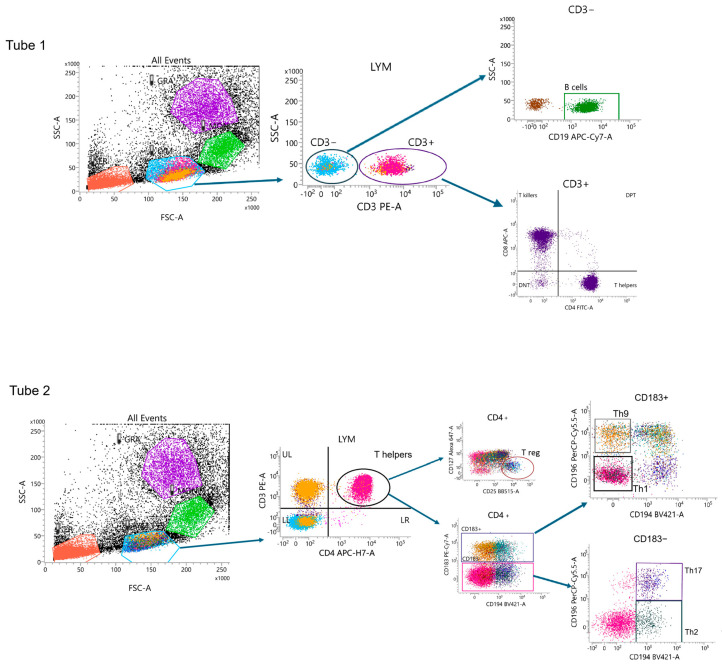
Representative gating strategy for the identification of the studied immune cell populations.

A broad flow cytometry panel was initially used to characterize multiple immune cell populations in early pregnancy, including total T cells, B cells, cytotoxic T cells, and several CD4^+^ T helper subsets (Figure A1). No significant differences were observed between pregnancy outcome groups in the frequencies of total T cells, B cells, cytotoxic T cells, T helper cells (Th), Th1, Th2, or regulatory T cells (*p* > 0.05; Table A1).

**Figure 3:**
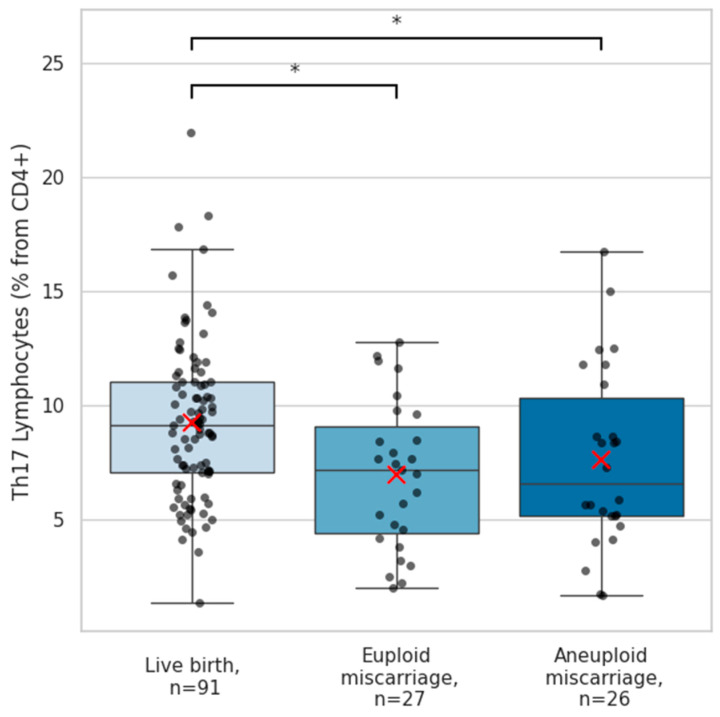
Quantities of Th17 cells according to reproductive outcome. Boxplots show circulating Th17 cell frequencies across women with subsequent live births (n = 91), euploid miscarriage (n = 27), and aneuploid miscarriage (n = 26). Th17 lymphocytes were identified as CD4^+^CD183^−^CD194^+^CD196^+^ cells using flow cytometry and quantified as a percentage of CD4^+^ cells. Individual data points are displayed as black dots, with red “×” symbols indicating group means. Group differences were evaluated using an independent-samples Kruskal–Wallis test (*p* = 0.005). Post hoc pairwise comparisons with Bonferroni correction demonstrated significantly higher Th17 cell levels in women with live births compared with those experiencing euploid miscarriage (*p* = 0.015) and in comparison with aneuploid miscarriage (*p* = 0.029). The asterisk “*” denotes significant difference between groups.

Among the immune populations analyzed, outcome-specific alterations were observed in Th17 and Th9 cells. The frequencies of pro-inflammatory Th17 lymphocytes in peripheral blood were significantly lower in patients who experienced miscarriage compared with those who had live births (median 7.1 [5.1]% vs. 9.0 [4.0]%; *p* = 0.001). This reduction was similar in euploid and aneuploid miscarriages, suggesting comparable Th17-cell responses in non-viable pregnancies regardless of fetal chromosomal status (Figure 3).

**Figure 4:**
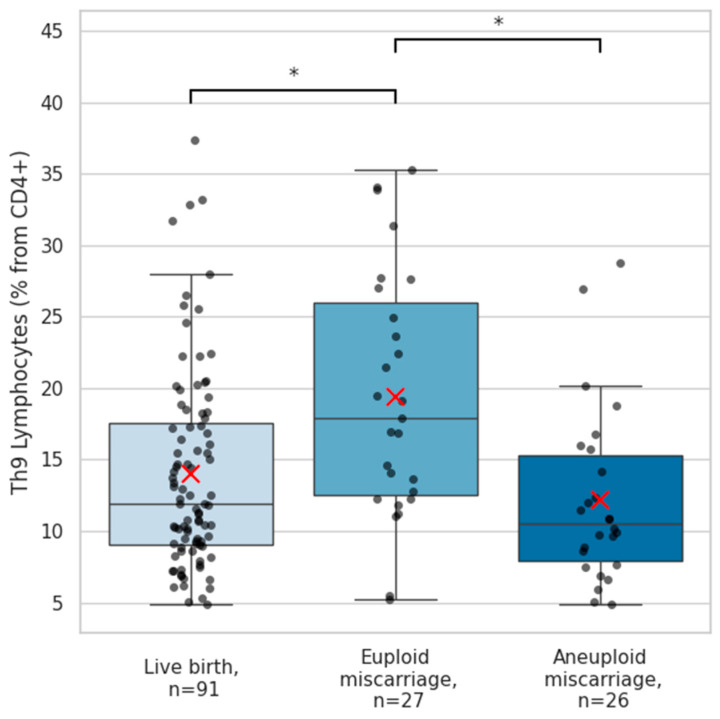
Quantities of Th9 lymphocytes according to reproductive outcome. Boxplots depict the quantities of circulating Th9 cells across women with subsequent live births (n = 91), euploid miscarriage (n = 27), and aneuploid miscarriage (n = 26). Th9 lymphocytes were identified as CD4^+^CD183^+^CD194^−^CD196^+^ cells using flow cytometry and quantified as a percentage of CD4^+^ cells. Individual data points are displayed as black dots, with red “×” symbols indicating group means. Group differences were evaluated using an independent-samples Kruskal–Wallis test (*p* = 0.002). Post hoc pairwise comparisons with Bonferroni correction demonstrated significantly higher Th9 cell levels in women experiencing euploid miscarriage compared with those with live births (*p* = 0.001) and in comparison with aneuploid miscarriage (*p* = 0.005). The asterisk “*” denotes significant difference between groups.

Another T-lymphocyte subset showing marked differences in early pregnancy was the Th9 population. Th9 cell frequencies were highest in pregnancies that subsequently resulted in euploid miscarriage (median 17.9 [14.8]%), distinguishing them from both aneuploid pregnancy losses (11.7 [8.2]%; *p* = 0.001) and normal live births (11.9 [8.7]%; *p* = 0.005) (Figure 4).

### 2.4. Discriminative Performance of Immune Markers Between Clinical Outcome Groups

**Figure 5:**
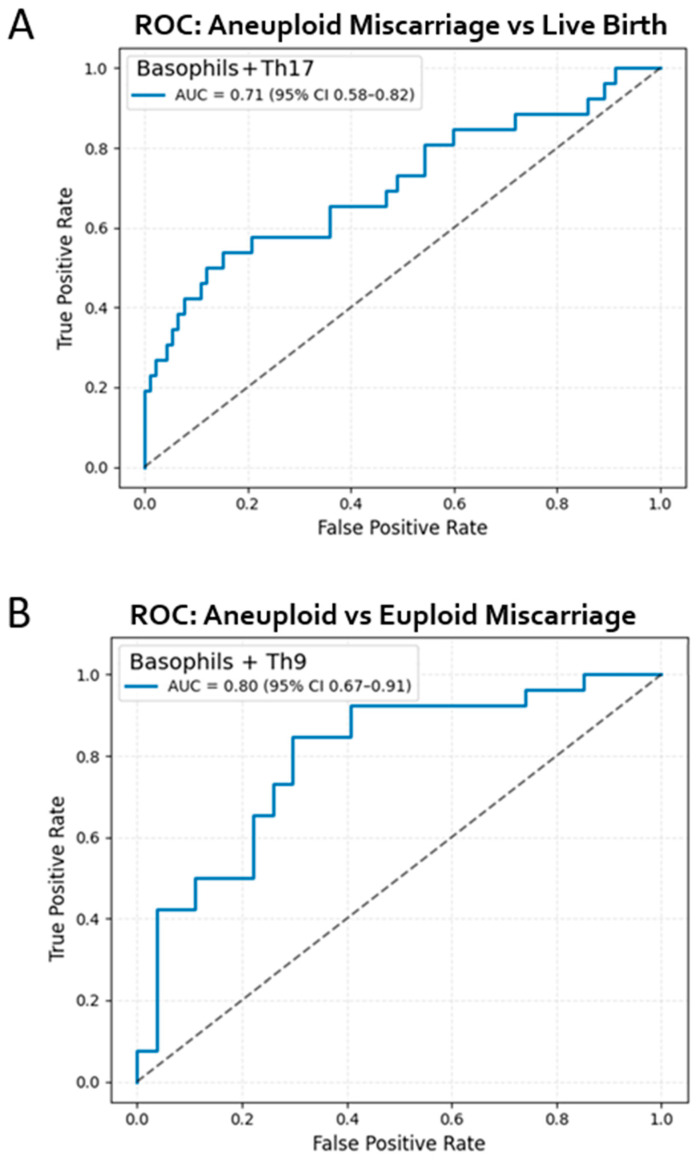
Receiver operating characteristic (ROC) curves for prediction of pregnancy outcome using peripheral immune cell quantities. The dotted diagonal line represents the performance of a random classifier (chance level, AUC = 0.5) (**A**) ROC curve illustrating the discriminative performance of a logistic regression model (AUC of 0.71 (95% CI 0.58–0.82)) incorporating Th17 and basophil levels to distinguish aneuploid miscarriage from live birth. (**B**) ROC curve for a logistic regression model distinguishing aneuploid versus euploid miscarriage using peripheral basophils and Th9 levels. The model demonstrated an AUC of 0.80 (95% CI 0.67–0.91).

To further evaluate the discriminative capacity of the immune parameters that differed significantly across outcome groups, receiver operating characteristic (ROC) analyses were performed. No single immune marker in our study was able to distinguish all three outcome groups (live birth, euploid miscarriage, and aneuploid miscarriage) on its own. In contrast, combinatorial marker analysis provided some discriminative power for differentiating two distinct pairs of outcomes. A logistic regression model incorporating Th17 and basophil levels was used to differentiate aneuploid miscarriage from live birth. This model demonstrated moderate discriminative accuracy, yielding an area under the curve (AUC) of 0.71 (95% CI 0.58–0.82), indicating that the combined Th17–basophil signature provides meaningful separation between viable pregnancies and losses attributed to chromosomal abnormalities (Figure 5A).

We next assessed whether immune parameters could distinguish between miscarriage subtypes. A model including basophils and Th9 cells showed good performance for differentiating aneuploid from euploid miscarriage, achieving an AUC of 0.80 (95% CI 0.67–0.91) (Figure 5B). These findings suggest that specific immune-cell patterns not only distinguish healthy pregnancies from those destined for loss, but also discriminate the underlying etiology of miscarriage.

Importantly, the ROC analyses were conducted on the same dataset used for the group comparisons, and therefore represent internal model performance. The results should be interpreted as exploratory indicators of discriminative potential rather than externally validated predictive tools.

These findings suggest a distinct maternal immune profile detectable in early gestation in pregnancies with chromosomally abnormal embryos.

## 3. Discussion

In this study, we identified distinct early peripheral immune signatures associated with pregnancy outcomes and demonstrated that specific combinations of circulating immune parameters can discriminate between live birth, euploid miscarriage, and aneuploid miscarriage. Although the immune alterations observed were modest in magnitude, their potential to classify pregnancy outcomes suggests a biologically meaningful interaction between early gestational immunoregulation and the chromosomal status of the developing embryo.

One of the clearest findings was the elevation of basophil counts in aneuploid pregnancy loss. Basophils are traditionally recognized for their roles in Th2 responses and allergic inflammation [17]. Their activation leads to the release of histamine and cytokines such as IL-4 and IL-13, which promote tissue vascularization and recruitment of other immune cells [18]. Murine single-cell data indicate they are also present at the maternal–fetal interface, suggesting a direct role in reproduction [19]. Their levels in the periphery are known to be relatively stable before, during and after pregnancy [20]. The markedly higher basophil concentrations observed in the blood of patients who later underwent an aneuploid miscarriage—particularly in pregnancies affected by trisomies, monosomies, and mosaicism—suggest that chromosomally abnormal embryos may provoke a systemic inflammatory or dysregulated innate immune response. The absence of basophil elevation in euploid miscarriage further supports the notion that this response is linked to embryonic chromosomal imbalance rather than to pregnancy failure. These results align with recent observations that embryonic abnormalities can elicit distinctive immune system perturbations. Recent evidence has shown differential expression of the C-C chemokine receptor type 10S and IFN gamma receptor in the serum of mothers carrying fetuses with trisomies 13 and 18, suggesting an impaired immune response in common aneuploidies [21]. While the specific role of basophils in pregnancy is not well established, they are usually involved in the recruitment and coordination of other immune cells that are well recognized to contribute to maternal–fetal tolerance and successful pregnancy. Nevertheless, the functional and clinical significance of basophil elevation in early pregnancy remains to be elucidated.

The next significantly altered immune cell population identified in our analysis was Th17 cells. Although classically pro-inflammatory, Th17 cells are known to play context-dependent roles in reproduction, including supporting angiogenesis, protecting the materno-fetal interface from extracellular microbes, and promoting trophoblast survival, proliferation, and invasion during early pregnancy via IL-17 secretion [22,23]. Decidual Th17 cells exhibit marked plasticity, and in successful pregnancies, they are often associated with Th2-type responses, with specialized Th17 populations producing IL-17, IL-4, and IL-22 localized to the implantation site [24,25]. In contrast, pathogenic Th17 phenotypes and excessive IL-17 signaling have been linked to adverse pregnancy outcomes such as recurrent implantation failure, recurrent miscarriage, pre-eclampsia, and preterm birth [14,26,27,28,29]. In our study, however, peripheral Th17-cell frequencies were reduced in both euploid and aneuploid spontaneous miscarriages compared with live births, with no differences observed between miscarriage subtypes. This uniform reduction suggests a generalized immune alteration in pregnancies destined to fail, rather than an effect driven by fetal chromosomal status, supporting the concept that both excessive and insufficient Th17 activity can disrupt normal pregnancy progression. It remains unclear whether the observed reduction in circulating Th17 cells represents insufficient inflammation or their redirection and trafficking toward the maternal–fetal interface in response to recognition of an unviable fetus.

Th9-cell quantities further distinguished euploid from aneuploid miscarriage, with the highest Th9 frequencies observed among euploid losses. These cells are typically associated with host–pathogen interaction, anti-tumor immunity, allergy and inflammation in autoimmune disorders via their production of IL-9 [30,31]. They are reported to be an early activated subpopulation of Th2 cells, although functionally and phenotypically distinct [32]. In the reproductive context, Th9 cells have been shown to regulate parturition timing in rodents; however, research on Th9 cells in human pregnancy is currently lacking [33]. It has been recently reported that circulating IL-9, whose primary source is Th9, is causally associated with hypertensive disorders of pregnancy [34]. Given that Th9 cells contribute to tissue repair and mucosal immunity [31,35], elevated Th9 quantities in patients experiencing euploid miscarriage may reflect an exaggerated or maladaptive compensatory immune response to an otherwise genetically normal failing pregnancy. Collectively, these observations highlight that the immunological landscape of miscarriage is heterogeneous and shaped not only by maternal factors but also by the intrinsic viability of the embryo.

The exploratory discriminative analyses reinforce these biological distinctions. A model integrating Th17 and basophil levels showed moderate separation between a live birth and aneuploid miscarriage (AUC 0.71), while basophils combined with Th9 cells more effectively distinguished aneuploid from euploid loss (AUC 0.80). Although these results should be interpreted cautiously due to the internal nature of the validation and the modest sample size, they demonstrate that even a small number of minimally invasive peripheral immune measurements may hold potential as early biomarkers of pregnancy viability and etiology. Importantly, these signals were detectable early in the first trimester (Table 1), underscoring the temporal proximity between embryonic development, maternal immune sensing, and pregnancy outcome.

Several limitations warrant consideration. The ROC analyses were conducted on the same dataset used for the discovery of group differences. External validation in an independent cohort is necessary before clinical application can be considered. Additionally, the immune parameters studied represent only a subset of the complex maternal immune environment. Future work integrating broader cytokine panels, decidual immune profiling, or multi-omics approaches may yield deeper mechanistic insights. Finally, although chromosomal status was determined by cytogenetic testing, the functional pathways linking specific aneuploidies to maternal immune activation remain incompletely understood.

Despite these limitations, our findings provide novel evidence that chromosomally abnormal embryos elicit a distinct maternal immune signature detectable in early pregnancy, and that specific peripheral immune-cell subsets may differentiate not only viable from failing pregnancies but also the underlying cause of miscarriage. It remains to be elucidated whether these immune changes represent a response to distinct stress signals released from the aneuploid fetus or a failing placenta drives inflammation. These results contribute to a growing body of work suggesting that early embryonic competence is reflected in, and potentially mediated by the maternal immune environment, with implications for early diagnostic strategies and for understanding the immunological determinants of reproductive success.

## 4. Materials and Methods

### 4.1. Study Design and Participants

**Figure 6:**
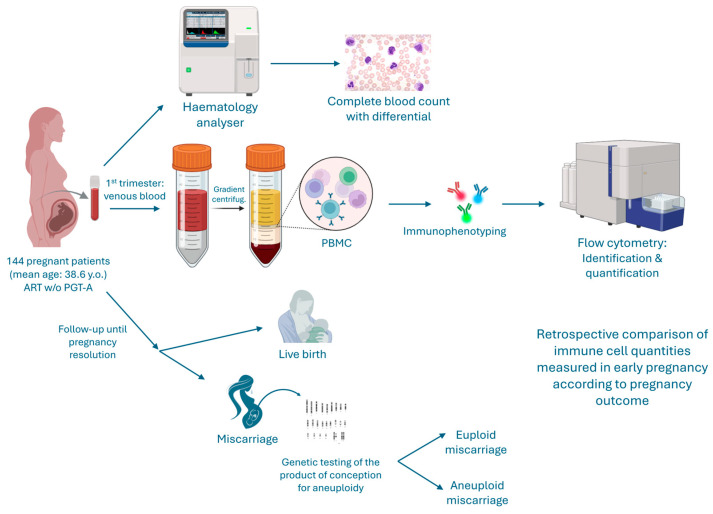
Experimental design. A total of 144 pregnant women (mean age 38.6 years) undergoing assisted reproductive technology (ART) without preimplantation genetic testing for aneuploidy (PGT-A) were enrolled during the first trimester (up to 12 weeks gestation). Peripheral venous blood samples were collected from each participant. A portion of whole blood was immediately processed using an automated hematology analyzer to determine complete blood count (CBC) with differential leukocyte counts. The remaining blood was subjected to density gradient centrifugation to isolate peripheral blood mononuclear cells (PBMCs), which were subsequently immunophenotyped by flow cytometry for identification and quantification of immune-cell subsets. Participants were followed prospectively until pregnancy resolution, classified as either live birth or miscarriage. Products of conception from miscarriage cases underwent genetic testing to determine ploidy status, distinguishing euploid from aneuploid losses. Retrospective analyses compared immune cell profiles measured in early pregnancy according to pregnancy outcomes. This workflow integrates clinical data with hematological and immunological assays to explore associations between immune cell populations during early pregnancy and pregnancy outcome.

This prospective observational study was conducted in accordance with the principles of the Declaration of Helsinki. Ethical approval was obtained from the Institutional Ethics Committee of Nadezhda Women’s Health Hospital (Approval No. 7A/13012023). All participants provided written informed consent prior to inclusion in the study. In total 144 women aged 23–47 years (mean ± SD, 38.6 ± 6.6 years) who achieved a confirmed intrauterine pregnancy following assisted reproduction (Figure 6) were enrolled. None of the included embryos underwent preimplantation genetic testing for aneuploidy (PGT-A). Inclusion criteria were: confirmed clinical pregnancy before 12 gestational weeks, availability of a peripheral blood sample, and willingness to continue follow-up to pregnancy resolution. Exclusion criteria comprised autoimmune disorders, allergic conditions, chronic or recent infections, systemic inflammatory diseases, recent vaccination, or immunomodulatory therapy within three months before conception.

### 4.2. Sample Collection and Processing

Peripheral venous blood (10 mL) was obtained from each participant during the first trimester (up to 12 weeks of gestation), using standard aseptic venipuncture procedures. Blood samples were collected into sterile K_2_EDTA-containing vacutainer tubes (ref: 367839, Becton Dickinson, Franklin Lakes, NJ, USA) to prevent coagulation and were gently inverted several times to ensure proper anticoagulant mixing. All samples were processed within two hours of collection and maintained at room temperature (20–22 °C) until processing. A portion of each blood sample was immediately analyzed using a Sysmex XN-1000™ hematology analyzer (Sysmex Corporation, Kobe, Japan) to determine the complete blood count (CBC), including erythrocyte indices, platelet count, total leukocyte count, and leukocyte differential. The analyzer was calibrated according to the manufacturer’s instructions and internal quality control procedures were performed daily. The remaining blood fraction was subjected to density gradient centrifugation to isolate peripheral blood mononuclear cells (PBMCs). Briefly, whole blood was diluted 1:1 with sterile phosphate-buffered saline (PBS) and carefully layered over human Pancoll (1.077 g/mL, PAN-Biotech GmbH, Aidenbach, Germany) in sterile 15 mL conical tubes. Samples were centrifuged at 400× *g* for 30 min at room temperature without brake. Following centrifugation, the PBMC layer located at the plasma–Pancoll interface was carefully aspirated using a sterile pipette and transferred to new tubes. The cells were then washed twice with PBS by centrifugation at 600× *g* for 5 min to remove residual plasma and platelets.

### 4.3. Flow Cytometry Immunophenotyping

Washed PBMCs were resuspended in PBS, and incubated with fluorochrome-conjugated monoclonal antibodies targeting surface markers of lymphocyte subsets for 15 min at 4 °C in the dark. The antibody panels included: CD3-PE (345765, BD Biosciences, San Jose, CA, USA), CD19-APC-Cy7 (348814, BD Biosciences), CD4–APC-H7 (560158, BD Biosciences, Heidelberg, Germany), CD4-FITC (345768, BD Biosciences), CD8-APC (555369, BD Biosciences), CD25–BB515 (564467, BD Biosciences), CD196 (CCR6)–BB700 (566477, BD Biosciences), CD183 (CXCR3)–PE-Cy7 (560831, BD Biosciences), CD127–Alexa Fluor 647 (558598, BD Biosciences), and CD194 (CCR4)–BV421 (562579, BD Biosciences). Samples were washed and immediately analyzed using a BD FACSLyric™ multiparameter flow cytometer. Gating strategy is available in Appendix A Figure A1. Data were processed using FACSuite™ v1.5.0.925 software (BD Biosciences).

The following immune cell populations were identified and quantified as percentages of total lymphocytes, total T lymphocytes, or T helpers, as appropriate: total T lymphocytes (CD3^+^), total B lymphocytes (CD3^−^CD19^+^), T helper cells (CD3^+^CD4^+^CD8^−^), cytotoxic T cells (CD3^+^CD4^−^CD8^+^), double-positive T cells (CD3^+^CD4^+^CD8^+^), double-negative T cells (CD3^+^CD4^−^CD8^−^), Th1 (CD4^+^CD183^+^CD194^−^CD196^−^), Th2 (CD4^+^CD183^−^CD194^+^CD196^−^), Th9 (CD4^+^CD183^+^CD194^−^CD196^+^), Th17 (CD4^+^CD183^−^CD194^+^CD196^+^), and regulatory T cells (CD4^+^CD8^−^CD25^+^CD127^−^).

### 4.4. Follow-Up and Outcome Classification

All participants were followed until pregnancy resolution. Pregnancy outcome was categorized into three groups—live birth, defined as delivery of a viable infant ≥37 weeks’ gestation, and aneuploid or euploid miscarriage, confirmed by chromosomal analysis of products of conception. Genetic testing of miscarriage tissue was performed using an NGS panel.

### 4.5. Statistical Analysis

Continuous variables were expressed as mean ± SD or median [interquartile range] based on data distribution. Comparisons between groups were conducted using Mann–Whitney U tests or Kruskal–Wallis tests for non-parametric data. A two-tailed *p* < 0.05 was considered statistically significant. Analyses were performed using SPSS v27.0 (IBM Corp, Armonk, NY, USA).

To evaluate the discriminative ability of the studied markers, exploratory receiver operating characteristic (ROC) analyses were conducted using logistic regression models incorporating immune quantities that differed significantly between the outcome groups. Predicted probabilities from each model were used to construct ROC curves and calculate the area under the curve (AUC) with corresponding 95% confidence intervals.

These analyses were performed to evaluate internal discriminative performance, and no external validation set was available; therefore, the results should be interpreted as exploratory.

## 5. Conclusions

In conclusion, early pregnancy complicated by chromosomal abnormalities is associated with distinct alterations in maternal peripheral immune cell composition. While Th17 levels are reduced in both euploid and aneuploid miscarriage, Th9 cells appear to be lower in aneuploid losses compared to euploid losses, and basophil counts are consistently elevated in aneuploid miscarriage cases. These findings suggest an early maternal immune response to genetically abnormal embryos and highlight that an integrated approach could inform the development of immune-based biomarkers for early risk assessment in pregnancy.

## Figures and Tables

**Table 1:** Patient and pregnancy characteristics according to reproductive outcome.

Characteristic	Live Birth (n = 91)	Euploid Miscarriage (n = 27)	Aneuploid Miscarriage (n = 26)	*p* Value
Age at pregnancy resolution, y	38.2 ± 6.9	39.8 ± 6.6	39.0 ± 6.0	NS
Gestational week (blood sample)	7.0 [3.0]	6.1 [1.0]	6.5 [1.2]	NS
Gestational week (pregnancy resolution)	39.1 [2.0]	8.0 [2.0]	8.0 [1.0]	0.02

## Data Availability

Data supporting reported results can be provided upon request from the corresponding author due to privacy restrictions.

## Supplementary Materials

The following supporting information can be downloaded at: https://www.mdpi.com/article/10.3390/ijms27062823/s1.

## Abbreviations

The following abbreviations are used in this manuscript:

ART	Assisted reproductive technologies
AUC	Area under the curve
β-hCG	Beta human chorionic gonadotropin
CBC	Complete blood count
CCR-	C–C chemokine receptor
CXCR-	C–X–C motif chemokine receptor
IFN	Interferon
IL-	Interleukin
IQR	Interquartile range
NGS	Next-generation sequencing
NIPT	Non-invasive prenatal testing
PBMCs	Peripheral blood mononuclear cells
PBS	Phosphate-buffered saline
PGT-A	Preimplantation genetic testing for aneuploidy
ROC	Receiver operating characteristic
SD	Standard deviation
Th	T helper
Treg	Regulatory T cells

## Appendix A

**Table A1:** Comparison of peripheral immune cell quantities according to pregnancy outcomes.

Immune Population, Unit	Live Birth (n = 91)	Euploid Miscarriage (n = 27)	Aneuploid Miscarriage (n = 26)	*p* Value
Leukocytes, ×10^9^/L	11.0 [8.1]	11.4 [8.2]	11.6 [6.8]	NS
Lymphocytes, ×10^9^/L	2.1 [1.0]	2.3 [1.2]	2.3 [0.9]	NS
Monocytes, ×10^9^/L	0.7 [0.3]	0.7 [0.4]	0.6 [0.3]	NS
Neutrophils, ×10^9^/L	8.0 [7.7]	8.9 [7.9]	8.8 [6.9]	NS
Eosinophils, ×10^9^/L	0.11 [0.13]	0.09 [0.14]	0.10 [0.15]	NS
Basophils, ×10^9^/L	0.05 [0.04]	0.05 [0.02]	0.09 [0.07]	0.009
Platelets, ×10^9^/L	229 [65]	233 [140]	245 [86]	NS
Total T cells/lymphocytes, %	72.7 [12.9]	72.1 [20.1]	67.8 [29.2]	NS
Total B cells/lymphocytes, %	5.5 [3.8]	5.3 [3.3]	5.9 [3.9]	NS
T helpers/total T cells, %	47.0 [14.0]	47.2 [22.8]	44.8 [20.4]	NS
Cytotoxic T/total T cells, %	34.8 [12.4]	35.9 [13.3]	37.1 [21.0]	NS
Th1/T helpers, %	16.5 [6.0]	15.6 [7.4]	16.3 [4.8]	NS
Th2/T helpers, %	6.7 [4.3]	7.0 [3.8]	8.3 [5.9]	NS
Th9/T helpers, %	11.9 [8.7]	17.9 [14.8]	11.7 [8.2]	0.005
Th17/T helpers, %	9.0 [4.0]	7.3 [5.7]	7.1 [5.1]	0.001
Treg/T helpers, %	7.3 [2.9]	6.9 [2.5]	7.3 [2.0]	NS

Data are presented as median [interquartile range]. *p* values were calculated using independent-samples Kruskal–Wallis tests.
